# Poor Law Topics

**Published:** 1905-11-11

**Authors:** 


### POOR LAW TOPICS.

### A USE FOR COUNTRY WORKHOUSES.

While pauperism as a whole has increased of late years, the increase has not been uniform nor universal. Poverty, like wealth, migrates, and places such as the parish of St. Giles, in London, which were once haunts of misery, show now a smaller rate of pauperism than formerly, chiefly because the old dens have been pulled down and replaced by offices and warehouses. In some parts of the country also, a diminished population has meant a diminished amount of pauperism, and in other districts a more generous indulgence in out-relief, while it does not lessen the number of paupers, but rather tends to increase them, leaves the workhouses emptier than of old. In other districts the workhouses are over full, yet to build new ones, in face of a constantly changing policy of administration of relief is unwise. Therefore it is customary in some unions to board out paupers in the comparatively empty workhouses of other places—a plan which profits both parties, saving the one the capital cost of building new institutions and the other the disproportionately high cost of management which is inevitable where a full staff must be provided for a small number of inmates. At a recent poor-law conference a speaker recommended a further development of this principle by suggesting that country workhouses in suitable localities might be used as convalescent homes for pauper invalids. This would be a great convenience. Although there is often very little social difference between the patients treated in hospitals and in some of our workhouse infirmaries, most convalescent homes refuse to receive within their walls those who are classed as paupers. Therefore boards of Guardians who wish to give the best possible chance either to convalescents who are leaving their infirmaries, or those who need a prolonged sojourn in better air than the neighbourhood affords, are compelled to pay comparatively large sums to those private institutions which take in poor-law patients. In these places the patients have practically complete independence. Between meal times they can come and go as they will; often they can pick up a few coppers which they spend at the public-house. It is not uncommon for them to appear in the police-court, and we can hardly believe that it is purely out of human kindness that the proprietor of the institution so often pays the fine inflicted on them. Many of these cases pass years of happy old age in these "homes," free from care of any sort, and placidly indifferent to the fact that they are costing the ratepayers of their Union anything from 12s. 6d. to 16s. a week. To send them to a country workhouse where, though arrangements were made for their obtaining whatever diet and rest they require, a discipline of some sort was exercised, would prevent a considerable amount of abuse of public money, and make many who now are content to doze away their life in idleness on a sunny beach, willing to return to their own homes.

